# Androgenic dependence of exophytic tumor growth in a transgenic mouse model of bladder cancer: a role for thrombospondin-1

**DOI:** 10.1186/1471-2490-8-7

**Published:** 2008-04-23

**Authors:** Aimee M Johnson, Mary J O'Connell, Hiroshi Miyamoto, Jiaoti Huang, Jorge L Yao, Edward M Messing, Jay E Reeder

**Affiliations:** 1 Department of Pathology and Laboratory Medicine, University of Rochester Medical Center, Elmwood Avenue, Rochester, New York, USA; 2 Department of Urology, University of Rochester Medical Center, Elmwood Avenue, Rochester, New York, USA; 3 Department of Imaging Science, University of Rochester Medical Center, Elmwood Avenue, Rochester, New York, USA

## Abstract

**Background:**

Steroid hormones influence mitogenic signaling pathways, apoptosis, and cell cycle checkpoints, and it has long been known that incidence of bladder cancer (BC) in men is several times greater than in women, a difference that cannot be attributed to environmental or lifestyle factors alone. Castration reduces incidence of chemically-induced BC in rodents. It is unclear if this effect is due to hormonal influences on activation/deactivation of carcinogens or a direct effect on urothelial cell proliferation or other malignant processes. We examined the effect of castration on BC growth in UPII-SV40T transgenic mice, which express SV40 T antigen specifically in urothelium and reliably develop BC. Furthermore, because BC growth in UPII-SV40T mice is exophytic, we speculated BC growth was dependent on angiogenesis and angiogenesis was, in turn, androgen responsive.

**Methods:**

Flat panel detector-based cone beam computed tomography (FPDCT) was used to longitudinally measure exophytic BC growth in UPII-SV40T male mice sham-operated, castrated, or castrated and supplemented with dihydrotestosterone (DHT). Human normal bladder and BC biopsies and mouse bladder were examined quantitatively for thrombospondin-1 (TSP1) protein expression.

**Results:**

Mice castrated at 24 weeks of age had decreased BC volumes at 32 weeks compared to intact mice (p = 0.0071) and castrated mice administered DHT (p = 0.0233; one-way ANOVA, JMP 6.0.3, SAS Institute, Inc.). Bladder cancer cell lines responded to DHT treatment with increased proliferation, regardless of androgen receptor expression levels. TSP1, an anti-angiogenic factor whose expression is inhibited by androgens, had decreased expression in bladders of UPII-SV40T mice compared to wild-type. Castration increased TSP1 levels in UPII-SV40T mice compared to intact mice. TSP1 protein expression was higher in 8 of 10 human bladder biopsies of normal versus malignant tissue from the same patients.

**Conclusion:**

FPDCT allows longitudinal monitoring of exophytic tumor growth in the UPII-SV40T model of BC that bypasses need for chemical carcinogens, which confound analysis of androgen effects. Androgens increase tumor cell growth *in vitro *and *in vivo *and decrease TSP1 expression, possibly explaining the therapeutic effect of castration. This effect may, in part, explain gender differences in BC incidence and implies anti-androgenic therapies may be effective in preventing and treating BC.

## Background

Racial and gender differences in bladder cancer (BC) incidence and survival were recently reviewed by Madeb and Messing [1]. BC is the fifth most common non-cutaneous cancer in the United States with over 67,000 newly diagnosed cases projected for 2007 [2]. Men are much more likely to be diagnosed with BC than women. Between 1992 and 2002, the incidence ratio for men relative to women by race ranged from 2.68 for African Americans to 4.26 for Native Americans. The male to female incidence ratio is remarkably consistent despite more than four-fold variation in incidence across race [3]. It is tempting to attribute the sexual incidence difference to environmental or lifestyle factors; however, the ratio has remained unchanged while women have entered the male workplace, and the incidence of smoking and smoking related diseases in females has increased considerably compared to that in men in most racial and demographic groups. It is unlikely that a purely environmental exposure etiology explains the differences between the genders in developing BC. A recent examination of BC risk in the United States Nurses Health Study cohort found an increased risk of BC in women associated with early age at menopause [4]. Of note is that many putative bladder carcinogens are activated or inactivated by cytochrome P450 enzymes, many of which are influenced by androgens [5-9].

Chemical carcinogenesis studies on mice have shown that males more readily develop BC than females, but that difference was nullified by castrating males. Testosterone given to castrated males decreased time to BC induction to rates of intact males [10]. In addition, the incidence of chemically-induced BC in male rats was increased by testosterone and decreased by estrogen supplementation [11]. Importantly, bladder carcinogenesis in rodents can be reduced by administration of luteinizing hormone-releasing hormone (LH-RH) agonists, the anti-androgen flutamide, and the 5-alpha reductase inhibitor finasteride [12]. More recently, androgen receptor (AR) knock-out (ARKO) and castrated male mice had a lower incidence of BC than wild-type male mice when given chemical carcinogens [13]. The gender and hormone dependent differences in chemically-induced BC might be attributed to sexual differences in activation or deactivation of carcinogens both in the bladder epithelium and in remote sites, or they might be due to sexual differences in regulation of a variety of relevant molecular processes such as cellular proliferation or response to DNA damage in the targeted tissue. Here we examine the question using a transgenic mouse model of BC that is not dependent on administration of carcinogens. The UPII-SV40T transgenic mouse model expresses the SV40 large T antigen specifically in the urothelium and reliably develops BC [14].

Thrombospondin-1 (TSP1) is a secreted protein that forms a 450-kDa homotrimer. Originally, TSP1 was identified as a component of platelet α granules [15,16], but TSP1 is now also known to be secreted by a variety of different cells including vascular smooth muscle [17], fibroblasts [18], and endothelial cells [19,20]. There are three different subgroups of thrombospondins: Subgroup A includes TSP1 and TSP2 which form homotrimers; Subgroup B includes TSP3, TSP4, and TSP5/COMP which form homopentamers [21]; and Subgroup C includes TSPs in insects which form pentamers but have distinct N-terminal domains [22]. Highly conserved among these subgroups is the carboxy end, which includes three EGF-like domains (called TSP Type 2 repeats), seven calcium-binding domains (TSP Type 3 repeats), and a globular C-terminal domain that is involved in cell binding. TSP1 and TSP2 also include, closer to the N-terminal end, three TSP Type 1 repeats (TSR, properdin), which have been implicated in signaling angiogenic inhibition through CD36 binding and support attachment of other cell types [[23,24]; see reviews]. The review by Adams and Lawler [25] summarizes that the angiogenic inhibitory action of TSP1 is mediated through inhibition of endothelial cell migration, induction of endothelial cell apoptosis, and inhibition of "growth factor mobilization and access to the endothelial cell surface."

Digestion of TSP1 with chymotrypsin results in several fragments, one of which is a 50 kDa N-terminal fragment that includes a procollagen homology domain and the Type 1 and Type 2 repeats [26]. This 50-kDa fragment was found to inhibit rat corneal neovascularization *in vivo *and endothelial cell migration *in vitro *[26]. Furthermore, active peptides derived from this 50 kDa fragment, from the procollagen and Type 1 repeat domains, were also found to block *in vivo *neovascularization [26]. These same TSP1 active peptides have been shown to bind to CD36, and neutralizing antibodies to CD36 were able to block native TSP1 and active peptide fragments from inhibition of endothelial cell migration [27]. Furthermore, TSP1 inhibition of corneal neovascularization was impaired in CD36 null animals [28]. In a review, Armstrong *et al*. [22] summarize a signal-transduction pathway in which a TSP1-stimulated CD36, an 88 kDa transmembrane glycoprotein, phosphorylates kinases p38 and JNK to induce transcription of FasL, which, when bound to Fas, leads to activation of caspases and results in apoptosis.

Low levels of TSP1 in human BC (by immunohistochemistry) were associated with low nuclear p53, increased tumor recurrence and progression, and decreased survival [29,30]. Cultured media from human BC cell lines stimulates migration and neovascularization and has decreased concentrations of TSP1 compared to media from normal urothelium, which actually inhibited these activities [31]. Additionally, androgens inhibit TSP1 expression in androgen-responsive tissues in rodents. Castrated male rats had increased expression of TSP1 and decreased microvessel density in the prostate, which could be reversed by administration of testosterone [32]. Treating mouse breast cancer cells with testosterone decreased *de novo *TSP1 synthesis (thus decreasing TSP1 expression at the mRNA and protein levels), and this effect was abrogated by administration of the anti-androgen flutamide [33], suggesting androgen-regulated angiogenesis. Here, we investigate the expression of TSP1 in human BC tissues and the influence of the androgenic milieu on TSP1 expression and BC growth in UPII-SV40T mice.

## Methods

### Animals

UPII-SV40T transgenic mice on FVB/N background (gift from Drs. Tung-Tien Sun and Xue-Ru Wu, New York University, NY) utilize the uroplakin II promoter to express simian virus 40 large T antigen specifically in the urothelium and reliably develop BC [14]. Animals were maintained in a pathogen-free facility where all care, experimental procedures, and euthanasia were performed in accordance with the policies set forth by the University Committee of Animal Resources at the University of Rochester Medical Center in compliance with federal guidelines.

### Genotyping

Tail biopsies were obtained from animals at weaning. DNA was extracted from the tails using an alkaline lysis solution (25 mM NaOH, 0.2 mM EDTA, pH 12.0) at 95°C for 1 hour followed by neutralization with an equal volume of 40 mM Tris-HCl at pH 5.0. The primer sequences for a ~ 500 bp SV40 T antigen product were GGACAAACCACAACTAGAATGCAGTG and CAGAGCAGAATTGTGGAGTGG. PCR cycling conditions were 94°C for 5 minutes followed by 12 cycles of 94°C for 20s, 64°C (decreasing by 0.5°C every cycle) for 30s, 72°C for 35s, then 25 cycles of 94°C for 20s, 58°C for 30s, 72°C for 30s, and a 10-minute final extension. Western blots for SV40 T in transgenic mouse bladders validated the PCR screening procedure (described in "Immunoblotting").

### Castration

UPII-SV40T transgenic male mice were randomized and either castrated (8 animals), castrated with the addition of a 60-day controlled release 1.5 mg dihydrotestosterone (DHT) pellet (Innovative Research of America, Sarasota, FL) (6 animals), or subjected to sham operations (9 animals) at 24 weeks of age after an initial flat panel detector-based cone beam computed tomography (FPDCT) scan. Wild-type and UPII-SV40T transgenic male mice were sham operated or castrated for analysis. Animals were administered 1 mg/ml acetaminophen *ad libitum *in drinking water for 24 hours prior to and following recovery surgery. Animals were anesthetized via isoflurane inhalation in an induction chamber and remained anesthetized throughout the procedure by using a nose-cone. Hair was removed from the scrotum with an electric shaver, and the area was swabbed with betadine followed by 70% ethanol. Gentle pressure was applied to the abdomen to cause the testes to descend into the scrotum. An axial incision of the scrotum was made midway between the anus and the penis. A small incision was made to the underlying fascia. Forceps were used to gently pull the testis through the incision. A slipknot of suitable resorbable suture material was placed around the vas deferens near the testis as a temporary tourniquet, which remained as an internal suture. The vas deferens was then cut between the suture and the testis, and the testis was removed. The incision was closed with suitable non-resorbable suture material in a simple interrupted pattern and again swabbed with betadine. Body temperature was maintained via the use of a warming pad. Animals were returned to cages and monitored until they could eat, drink, and ambulate normally. Sham operations included all physical manipulations except the internal sutures and removal of the testes.

### Flat panel-based cone beam computed tomography

UPII-SV40T transgenic mice were administered tail vein injections of 0.15 cc Omnipaque™ (Iohexol), anesthetized via isoflurane inhalation for 15 minutes, and scanned as previously described [34-36] at 24, 28, and 32 weeks of age. The 290 2D images acquired in each 10-second 360° scan were reconstructed by a filtered back projection-based modified Feldkamp algorithm and analyzed with Amira, version 3.1.1-1, for MacOSX [36]. In addition, reconstructed data sets were also visualized and tumor volumes calculated in ImageJ 1.36b (NIH, USA). Tumor volumes were calculated from the difference in areas in each slice after thresholding images to select the bladder area and the Omnipaque™-filled area. Mean tumor volumes between groups were compared using one-way ANOVA and t-test (JMP 6.0.3, SAS Institute Inc., Cary, NC).

### Immunohistochemistry

Five μm sections of formalin-fixed, paraffin-embedded mouse bladders from intact, castrate, and castrate + DHT UPII-SV40T male mice at 32 weeks of age were mounted and dried on glass slides. The slides were dried, baked at 57°C overnight, and deparaffinized. Endogenous peroxidase activity was quenched by incubation in 0.3% hydrogen peroxide in PBS for 30 minutes. Antigen unmasking was performed by 25-minute microwave incubation in 10 mM citrate buffer (pH 6.0). Slides were incubated at room temperature for one hour with: rat anti-CD34 (1:50; Abcam 8158, Cambridge, MA), rabbit anti-SV40T (1:100; Santa Cruz 20800, Santa Cruz, CA), mouse anti-TSP1 (1:400; Neomarkers Ab-4, Clone A6.1, Fremont, CA), or rabbit anti-AR (1:100; Santa Cruz 815). Secondary antibodies were peroxidase conjugated. CD34, SV40T, and AR were detected with DAB (Dako, Carpinteria, CA) for 10 minutes. TSP1 was detected with AEC+ (Dako) for 10 minutes. Slides were then counterstained in Modified Mayer's Hematoxylin, blued in 3% ammonia, rinsed in tap water, and mounted. Hematoxylin/eosin slides were also prepared. Slides were viewed at 100× or 200× original magnification with a light microscope.

Five μm sections of formalin-fixed, paraffin-embedded urothelial biopsies from ten male cancer patients (9 high-grade T2–T4, 4 with lymph node involvement, 5 with metastases; 1 low-grade Ta, with lymph node involvement) with foci of benign urothelium were mounted and dried on chemically charged slides, followed by baking at 57°C overnight, deparaffinization, and quenching of endogenous peroxidase activity by incubating in 3% hydrogen peroxide for 6 minutes. The peroxide was cleared from the slides by rinsing in running water then TBS (50 mM Tris-HCl, 150 mM NaCl, 0.05% Tween-20, pH 7.6). Antigens were unmasked by 12-minute heat retrieval in citrate buffer, pH 6.1 (Dako) using the Borg Decloaking Chamber (Biocare Medical, Walnut Creek, CA) set to 120–123°C, 18–21 psi. After antigen unmasking, slides were allowed to cool for 15 minutes prior to staining, rinsed in TBS for 5 minutes, and placed on a DAKO autostainer. Slides were exposed at room temperature to mouse monoclonal anti-AR antibody (Dako, clone AR441, 1:25) for 60 minutes, Horse Anti-Mouse IgG Biotin (Vector Laboratories, Burlingame, CA) for 30 minutes, and Streptavidin-HRP (Jackson Labs, West Grove, PA) for 30 minutes. A 10-minute AEC+ (Dako) incubation was used to develop slides, followed by rinsing in distilled water, counterstaining in Modified Mayer's Hematoxylin, bluing in 3% ammonia, rinsing in tap water, and mounting with aqueous mounting media. Slides were viewed at 400× original magnification with a light microscope.

### Mouse bladder cancer explant

A tumor-bearing male UPII-SV40T (MMUST) transgenic animal was sacrificed, and the entire bladder was removed intact and placed in sterile PBS. The bladder was rinsed in sterile PBS before transferring to sterile DMEM. In DMEM, the bladder was bisected, and the urothelium (inner layer of the bladder) was scraped out with a scalpel, finely minced, and cultured in DMEM supplemented with 10% FBS, 2 mM L-glutamine, and penicillin (100 u/ml)/streptomycin (100 μg/ml), 2.5 μg/ml fungizone in a humid 5% CO_2 _chamber at 37°C. This explant was maintained in culture for six months. RT-PCR for cytokeratins 13 and 20 were performed to, respectively, confirm transitional-type and terminally differentiated urothelium [37].

### Tissue culture

Human prostate cancer (LNCaP, PC3; ATCC, Manassas, VA), human BC (J82-male, TCCSUP-female; ATCC), and UPII-SV40T bladder cancer explant (MMUST) were maintained in 10% FBS and assayed in 5% charcoal-stripped, dextran-treated FBS at 37°C in phenol red-free DMEM 2 mM L-glutamine, and penicillin (100 u/ml)/streptomycin (100 μg/ml) in a humid 5% CO_2 _incubator.

### Cell proliferation assay

Cells were cultured in 12-well plates with the addition of ethanol (0.001%) or DHT in ethanol vehicle at final concentration of 1 nM or 10 nM DHT for 72 hours with media change and treatment replenishment after 48 hours. After 72 hours of DHT or ethanol treatment, 100 μl of 3-(4,5-Dimethylthiazol-2-yl)-2,5-diphenyltetrazolium bromide (MTT, Sigma) stock solution (5 mg/ml) was added to each 1 ml media in the wells and incubated at 37°C for three hours, followed by addition of 1 ml of 0.04N HCl in isopropanol at room temperature with agitation for 15 minutes. Absorbance was read at 570 nm with a background absorbance at 660 nm. Experiments were performed in triplicate, and mean absorbance ± the standard error was calculated in Microsoft^® ^Excel (Microsoft Corporation, Redmond, WA).

### Immunoblotting

Whole bladders and a panel of organs from 20–24 week-old mice and frozen biopsies of human normal bladder and cancer (paired from the same patient) were homogenized in 1× RIPA lysis buffer with added protease inhibitors (Sigma, P8340, St. Louis, MO). Human samples (different than those used for IHC) were obtained from 10 males ranging in age from 60–81 years. All patients had high grade, invasive urothelial carcinomas (3 metastatic), and samples were obtained with informed consent and University of Rochester Research Subject Review Board approval of the protocol. Normal urothelium and BC specimens were identified by experimental urologists and genitourinary pathologists.

Cell cultures were scraped in PBS, centrifuged, and resuspended in 1× RIPA lysis buffer with added protease inhibitors. Proteins were separated by SDS-PAGE on 7.5% acrylamide gels. Proteins were transferred to a nitrocellulose membrane, blocked overnight at 4°C in 5% milk, incubated with goat anti-human N-terminal TSP1 (diluted 1:200; Santa Cruz 12312), rabbit anti-AR (diluted 1:200; Santa Cruz 815; cross-reacts with mouse and human), mouse anti-AR (diluted 1:200; Dako, clone AR441; cross-reacts with human only), or rabbit anti-SV40 T (diluted 1:200; Santa Cruz 20800) antibodies for one hour and horseradish-peroxidase labeled secondary antibodies for one hour, and visualized by chemiluminescent detection in Western Lightning (Perkin-Elmer, Boston, MA) and exposure to X-omat or BioMax Film (Kodak, Rochester, NY). Developed films were placed on transmitted white light, and then images were captured using a Q Imaging CCD camera and processed using NIH ImageJ.

## Results

### Total bladder cancer volume determined by flat panel detector-based cone beam computed tomography

**Figure 1:**
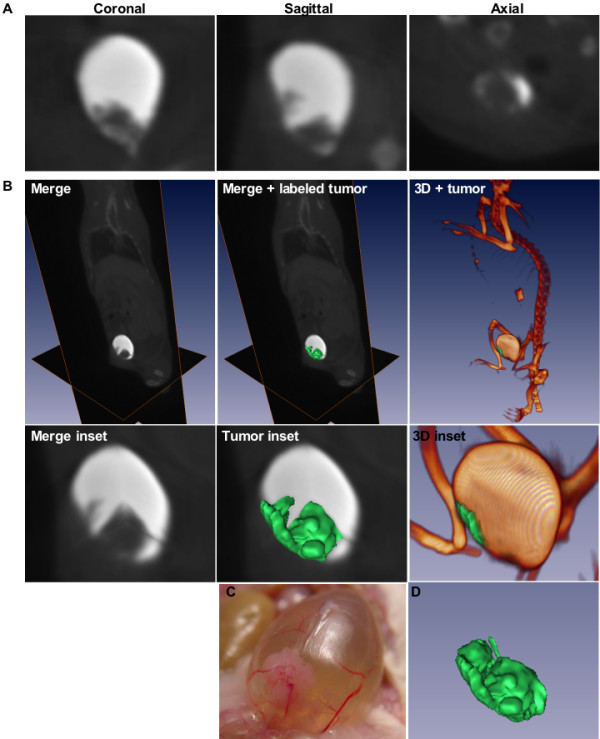
**FPDCT scans, 3-dimensional reconstruction, and necropsy photo of UPII-SV40T transgenic mouse**. (*A*) Coronal, sagittal, and axial CT sections of a bladder with exophytic tumor. (*B*) Overlay of coronal, sagittal, and axial CT slices; overlaid CT slices with green pseudo-colored tumor; 3D rendering of high contrast areas – skeleton and contrast-filled bladder – with green pseudo-colored exophytic tumor. Insets: Magnified bladder sections of CT overlay, overlay with pseudo-colored tumor, and 3D rendering of high contrast areas with pseudo-colored tumor, respectively. (*C*) A large exophytic bladder tumor (urothelial opacity) fed with an extensive network of blood vessels is visualized by backlighting the bladder. (*D*) 3D pseudo-colored bladder tumor only.

To monitor BC volume in male UPII-SV40T mice, animals were scanned using an FPDCT scanner at three different times points four weeks apart. Coronal, sagittal, and axial scans of UPII-SV40T animals depicted bladders filled with contrast agent and allowed visualization of tumors, noted as filling defects (Fig. 1A). Three-dimensional reconstruction of the FPDCT scans with pseudo-colored tumors overlaid indicated the exophytic nature of the BC (Fig. 1B, D). At necropsy, it was noted that large exophytic mouse BCs (epithelial opacity, Fig. 1C) were fed by extensive vascular networks grossly visible though the bladder wall.

**Figure 2:**
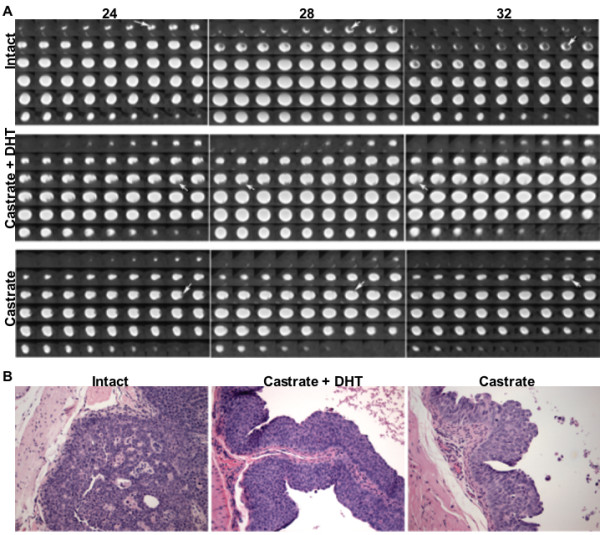
**CT and histological sections of mouse bladders with space-filling tumors**. (*A*) Sequential whole body axial slices from FPDCT scans taken at three time points (24, 28, and 32 weeks of age) were cropped to select the bladder, with the lumen filled with a radiopaque agent and appearing lighter than the surrounding tissue. Areas of radiopaque exclusion in the lumen are indicated with arrows. Animals were either sham-operated, castrated + DHT pellet, or castrated within one week after the initial 24-week FPDCT scan. Tumor volumes were calculated from the difference in area in each slice after thresholding to select the bladder area and the Omnipaque™-filled area. (*B*) Hematoxylin/eosin stained sections of bladders from UPII-SV40T male intact, castrate + DHT pellet, and castrate mice. 100× original magnification.

A montage of axial slices through the bladder illustrates the presence of contrast agent in the bladder as a positive image on a mostly negative field (Fig. 2A). Exophytic tumors protruding into the lumen from the bladder wall were visible beginning at 24 weeks of age in the UPII-SV40T mice (arrows, Fig. 2A). Filling defects were not observed at 16 and 20 weeks, although carcinoma in situ is present in UPII-SV40T mice as early as one month [14]. Histological analysis of UPII-SV40T bladders shows the exophytic tumor growth (Fig. 2B). Castration changes the morphology of bladder tumors compared to the tumors seen in intact animals, but urothelial dysplasia is persistent in castrated mice (Fig. 2B).

**Figure 3:**
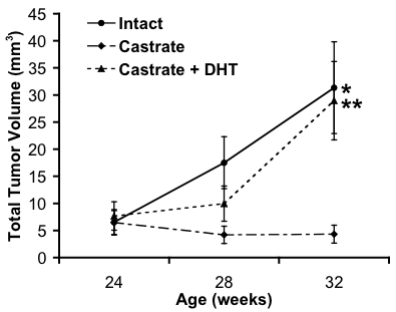
**Bladder cancer growth in UPII-SV40T transgenic male mice**. Animals were anesthetized, injected with radiopaque agent via tail-vein, and submitted to FPDCT scanning for a total of 3 longitudinal sessions. Data sets were analyzed with Amira and NIH ImageJ to identify tumors and quantify tumor volumes. Tumor volumes for intact (n = 9), castrate (n = 8), castrate + DHT (n = 6) are indicated. Values are mean ± SE. p = 0.0071*, p = 0.0233** vs. castrate (one-way ANOVA, JMP 6.0.3, SAS Institute, Inc.).

In intact and castrate + DHT animals, individual tumors continued to grow larger in an exophytic manner over an eight-week period while tumors ceased exophytic growth in animals castrated at 24 weeks (Fig. 2A, 3). In fact, tumors in 5 of 8 castrated animals decreased in size. Castrated animals implanted with slow-release DHT pellets appeared to have slightly slowed tumor growth between the castration/pellet implantation at 24 weeks and the following FPDCT scan at 28 weeks, but tumor growth continued 4 weeks after castration and DHT implantation (Fig. 3). At 24 weeks (immediately before sham, castration, and castration/DHT implantation procedures), mean tumor volumes were 6.5 ± 2.3, 6.5 ± 2.2, and 7.7 ± 2.6 mm^3^, respectively. At 32 weeks, mean tumor volumes were 31.4 ± 8.4 mm^3 ^for 9 intact animals, 4.3 ± 1.7 mm^3 ^for 8 castrated animals, and 28.9 ± 7.2 mm^3 ^for 6 castrate/DHT animals. Intact and castrate/DHT mean tumor volumes at 32 weeks were not different, p = 0.8055 (one-way ANOVA, JMP 6.0.3, SAS Institute, Inc.); however, castrate mean tumor volumes were significantly different from intact, p = 0.0071, and castrate/DHT, p = 0.0233.

### Expression of androgen receptor in human urothelium and bladder cancer

**Figure 4:**
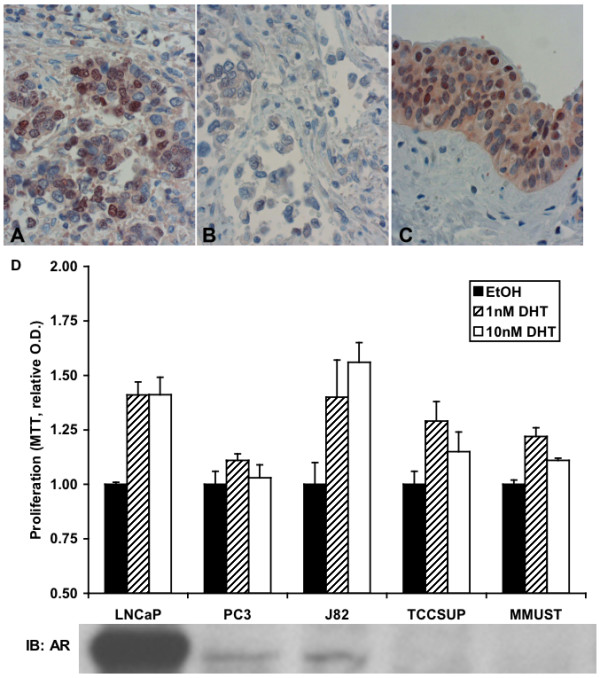
**AR expression and DHT response in bladder**. Nuclear AR expression (brown-stained cells) in (*A, B*) human bladder cancer and (*C*) benign urothelium at 400× original magnification. Dako antibody clone AR441 was used for labelling. (*D*) MTT assay of human prostate cell lines (LNCaP and PC3), human bladder cancer cell lines (J82 and TCCSUP), and male mouse urothelial explant from UPII-SV40T animal (MMUST). Cells were treated with ethanol or DHT in ethanol vehicle for 72 hours prior to MTT assay. Experiments done in triplicate. Values are relative means ± SE. Western blot analysis using Santa Cruz 815 antibody was performed to determine AR expression.

Of the ten human BC samples examined, BC showed 50% positive staining for the AR (Fig. 4A, B). There were 15 foci of benign urothelium included on these slides; 10 were positive and 5 were negative for AR. The positively stained benign urothelium had nuclear staining for the AR in cells above the basal layer (Fig. 4C).

### Dihydrotestosterone stimulates proliferation of bladder cancer cells *in vitro*

LNCaP, a high AR-expressing androgen responsive prostate cancer cell line, and PC3, a low AR-expressing androgen-independent prostate cancer cell line, were respectively used as positive and negative controls for DHT-stimulated cell proliferation. Human BC cell lines TCCSUP and J82 treated with DHT had increased cell proliferation compared to controls, despite neither cell line expressing very much or any AR (Fig. 4D). Mouse BC explant MMUST from a male UPII-SV40T also responded to DHT treatment with an increase in cell proliferation, again despite a lack of AR protein expression (Fig. 4D).

### Microvessel and thrombospondin-1 distribution in UPII-SV40T bladder

**Figure 5:**
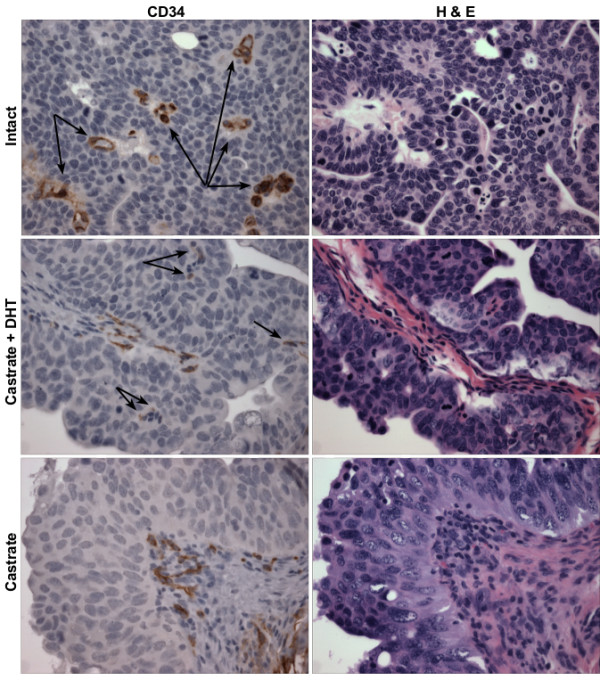
**CD34 microvessel staining in mouse bladder**. Membrane expression of CD34 (brown staining) indicates the presence of microvessels. Male UPII-SV40T animals were sham-operated (intact), castrated and administered DHT, or castrated at 24 weeks of age. Animals were euthanized and bladders harvested at 32 weeks of age. Tissue was formalin fixed and paraffin embedded. Examples of positive CD34 staining in the tumor urothelium are indicated with arrows. CD34 staining is also present in the stroma, corroborated by adjacent HE stained sections. Magnification: 200× original.

**Figure 6:**
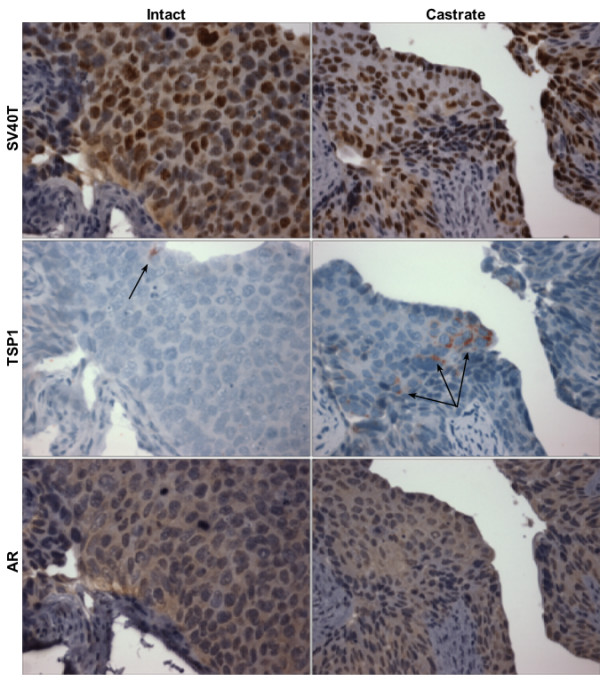
**Immunohistochemical staining of mouse bladder**. Male UPII-SV40T animals were sham-operated (intact) or castrated at 24 weeks of age, and then animals were sacrificed at 32 weeks and bladders harvested, formalin-fixed, and paraffin embedded. The upper panel shows similar levels of SV40T staining in the urothelium of both intact and castrated animals. TSP1 expression is indicated by arrows in the center panels. AR is also expressed in the urothelium of both intact and castrated animals (anti-AR, Santa Cruz 815). Magnification: 200× original.

Since the exophytic nature of the UPII-SV40T BC suggests a role for angiogenesis, 5 μm sections of UPII-SV40T mouse bladders at 32 weeks of age were stained for CD34, a marker of microvessels, or TSP1. Animals had been either sham-operated, castrated and administered DHT, or castrated at 24 weeks of age. CD34 was found to be present in the stroma as well as in the BC of sham-operated and castrated + DHT animals. Castrated animals expressed CD34 only in the stroma (Figure 5). Secreted TSP1 was found to be expressed in the bladder stroma of all animals, but TSP1 expression in the urothelium was greater in castrated animals when compared to intact animals (Figure 6). Staining for SV40T was performed to confirm that *uroplakin II *promoter was not androgen responsive: SV40T is present in the urothelial cells of both intact and castrated animals (Figure 6). Additional immunohistochemical staining for AR confirmed the urothelial presence of AR in both intact and castrated animals (Figure 6).

### Thrombospondin-1 expression in normal bladder and tumor

**Figure 7:**
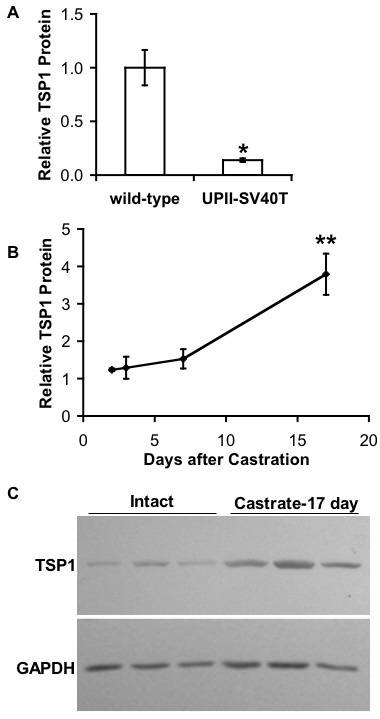
**TSP1 protein expression in mouse bladder**. Whole bladder lysates of sham-operated (intact) wild-type and sham-operated (intact) or castrated UPII-SV40T male mice were assayed and quantified for TSP1 expression by western blot analysis. (*A*) Relative bladder TSP1 protein expression in wild-type vs. UPII-SV40T mice. Values are relative means ± SE. For each group, n = 3. p = 0.0326*. (*B*) Relative bladder TSP1 protein expression in UPII-SV40T mice sacrificed 2, 3, 7, and 17 days after castration. Intact relative expression = 1. Values are relative means ± SE. For each group, n = 3. p = 0.0154** vs. intact. (*C*) Western blot of intact (n = 3) vs. 17-day castrate (n = 3) whole bladders from UPII-SV40T mice. TSP1 immunoblots were quantified. GAPDH control blot verified equal loading. P values determined by paired, two-tailed, student's t-test.

Proteins from whole male mouse bladders (20–24 weeks of age) were subjected to western blot analysis. The molecular weight of TSP1 expressed in mouse and human bladders was slightly less than 50 kDa. Wild-type mouse expressed significantly more TSP1 than transgenic UPII-SV40T in the bladder (p = 0.0326, Fig. 7A; paired, two-tailed, student's t-test). Castration of UPII-SV40T male animals resulted in a modest (but not statistically significant) increase in TSP1 expression in bladder protein at days 2–7 post-castration compared to their intact counterparts (Fig. 7B). Seventeen days after castration in UPII-SV40T, TSP1 bladder protein expression was increased 3.8-fold (p = 0.0154) compared to intact animals (Fig. 7B, C). GAPDH immunoblot controls verified equal loading of samples (Fig. 7C).

**Figure 8:**
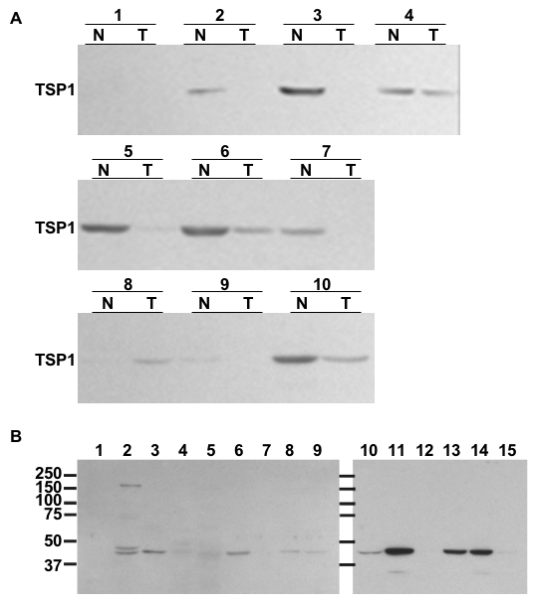
**TSP1 protein expression in human bladder and mouse tissues**. (*A*) Protein lysates from paired human normal (N) and tumor (T) bladder tissues were also immunoblotted for TSP1. (*B*) Tissue protein lysates from mouse 1) skeletal muscle, 2) brain, 3) lung, 4) pancreas, 5) liver, 6) spleen, 7) heart, 8) intestine, 9) skin, 10) testes, 11) prostate, 12) seminal vesicle, 13) ovary, 14) uterus, 15) kidney.

Paired normal and BC frozen biopsies from ten male patients were also examined for expression of TSP1 protein by western blot analysis. TSP1 expression was greater in 8 (specimens 2, 3, 4, 5, 6, 7, 9, 10; Fig. 8A) of the normal bladder specimens compared to the paired BC specimen. One tumor/normal tissue pair showed no detectable TSP1 at all (specimen 1), while another tumor/normal tissue pair (specimen 8) showed greater TSP1 expression in the tumor compared to the normal tissue (Fig. 8A).

The hormonal status of these patients was unknown. AR protein expression was evaluated in these samples by western blot analysis (anti-AR, Dako clone AR441). AR expression was higher in patient samples in which TSP1 expression was higher, except in specimen 1 where AR expression was actually higher in the tumor sample and TSP1 was not detected in either the normal or BC sample (data not shown). However, among the patient specimens, TSP1 expression was significantly greater in normal tissue than in BC (p = 0.009; paired, two-tailed, student's t-test) while AR expression was not significantly different (p = 0.125). GAPDH loading controls verified equal loading of protein samples (data not shown).

A panel of mouse tissue lysates was investigated for TSP1 expression (Fig. 8B). Brain, lung, pancreas, liver, spleen, intestine, skin, testes, prostate, ovary, uterus, and kidney (lanes 2, 3, 4, 5, 6, 8, 9, 10, 11, 13, 14, 15, respectively) all had detectable levels of a TSP1 fragment slightly smaller than 50 kDa. Brain (lane 2) also expressed TSP1 fragments of ~ 140 kDa, 70 kDa, and a doublet present between 37–50 kDa. Pancreas (lane 4) also expressed this doublet band between 37–50 kDa. Only prostate and uterus (lanes 11 and 14, respectively) expressed an even smaller TSP1 fragment less than 37 kDa. TSP1 expression in mouse bladder is shown in Figure 5C and was not included in this tissue panel. The ~ 450 kDa trimeric form of TSP1 was not detectable on a gradient gel under denaturing conditions (data not shown).

## Discussion

Non-invasive *in vivo *imaging of small animals minimizes the number of animals used in experiments, increases the information about real-time phenomena unlike post-mortem analyses, and demonstrates the effects of interventions within a given host. Currently, however, many *in vivo *imaging instruments function on an image acquisition time-scale of 5 minutes, sufficient time for animal movement resulting in blurred reconstructed data sets, to hours, at which point animal recovery and longitudinal studies become difficult. Considering the implications of using conventional and commercially available *in vivo *imaging techniques on animal welfare and longitudinal studies, we utilized a unique FPDCT instrument that can acquire 290 2D images in 10 seconds [34]. With this instrument, multiple imaging sessions of individual animals are possible, permitting the monitoring of BC growth in 23 animals presented here. Through the intravenous administration of Omnipaque^®^, a radiopaque agent which is filtered from the bloodstream by the kidneys, excreted into urine, and collected in the bladder, FPDCT scans allowed measurement of exophytic BC, which were defined as areas of contrast exclusion (Fig. 1A, B). Tumor volumes were quantifiable via image analysis software used to label the exophytic tumors (contrast-excluded areas; Figure 1B, D).

We observed that exophytic growth of mouse BC was decreased by castration and restored by DHT administration. Mean tumor volumes for intact and castrate/DHT animals were not different from each other but were significantly different from the mean tumor volume of castrate animals. This finding indicates that surgical castration is sufficient to partially overcome the strong tumorigenic drive of SV40 large T antigen. The UPII-SV40T transgenic animal reliably produces BC that mimics BC in humans [14] and is free from complications associated with BC models using chemical carcinogens, which are metabolized by an androgen-influenced P450 system in the liver and urothelium [5-9]. It has long been hypothesized that sexual differences in steroid hormones or their receptors may contribute to the difference in BC incidence between the genders in humans and animals, although previous *in vivo *studies of steroid hormones and BC have relied on chemical carcinogen models [10-13].

With regard to the sexual difference in the incidence of BC, AR expression has been investigated in bladder tissue. AR is expressed in normal mouse [38], rat [39-41], and human [42] bladder epithelia as well as human BC cell lines [13,43]. Human BC biopsies from males and females had a greater concentration of AR than normal human urothelium [44], indicating that AR might be useful as a diagnostic marker for BC. However, the same study [44] also showed that the AR levels decreased with increasing tumor grade in BC, which has been corroborated by steroid binding [45] and immunohistochemical [46] studies. This decreased expression of AR in higher grade tumors may indicate a more poorly differentiated cell type or, as in prostate cancer, a transition to an androgen-independent state. In addition, it should be noted that experiments by Guerini *et al*. [47] indicate that steroid hormone receptors other than AR may be activated by DHT and its metabolites. As such, we investigated the *in vitro *growth of BC cell lines and UPII-SV40T urothelial explants, which express little or no AR, and found that DHT was still able to increase proliferation. We were also able to detect AR in the bladders of intact and castrated UPII-SV40T mice but did not notice a significant difference in the expression levels. Furthermore, Miyamoto *et al*. [13] showed that DHT treatment of BC cell lines stimulated proliferation *in vitro*, and this effect was abrogated by the anti-androgen hydroxyflutamide; ARKO male mice treated with BBN did not develop BC, but 25% of BBN-induced ARKO males developed BC. Taken together, Miyamoto's data suggest the potential for two separate mechanisms: 1) an androgen-mediated, classical AR signaling pathway, and 2) an androgen-regulated, non-AR dependent signaling mechanism in BC. The data presented here support the latter mechanism without contradicting the possibility of a classical AR-dependent pathway.

We speculated that the exophytic growth might be dependent on neo-vascularization. Because there were such significant effects on the morphology of the bladder tumors, microvessel density counts could not be performed in a systematic manner; bladder tumors could not be compared to normalized urothelium. However, CD34 expression was noted in the bladder stroma and BC urothelium of intact and castrate + DHT animals whereas CD34 was only present in the bladder stroma of castrated animals. The absence of CD34 staining for microvessels in the BC of castrated animals indicates that there may be less angiogenesis in castrated animals compared to their intact and DHT augmented counterparts. Additionally, the anti-angiogenic factor, TSP1, which plays a strong angiogenic-inhibitory role and whose expression is suppressed by androgens, was investigated. The TSP1 promoter has been shown to contain a hormone response element that is sensitive to testosterone agonist administration [32]. In addition, the same study also found that castration of rats increases TSP1 expression and decreases microvessel density counts in the prostate [32]. Metastatic prostate cancer patients treated with androgen deprivation therapy expressed a significantly higher TSP1 immunoreactivity in prostatic adenocarcinoma when compared to other metastatic prostate cancer patients who did not receive hormonal therapy [48]. Testosterone administered *in vitro *to breast cancer cells has been shown to decrease TSP1 expression at the mRNA and protein levels, and this effect can be inhibited by pre-incubation with the anti-androgen flutamide [33]. Western blot analysis of intact male mouse bladders indicated that wild-type animals expressed more of the ~ 50 kDa TSP1 fragment (previously shown to have a neovascularization inhibitory role *in vivo*) [26] than their tumor-bearing UPII-SV40T transgenic counterparts, supporting an anti-angiogenic role for TSP1 in the exophytic growth of UPII-SV40T BC. Furthermore, castration increased the TSP1 protein expression in both wild-type and transgenic animal bladders. It is interesting to note that mouse uterus expressed the ~ 25 kDa fragment of TSP1, which has previously been shown to have pro-migratory and neovascularization effects [26]. This neovascularization fragment of TSP1 may be of use to the uterus during the estrus cycle by providing a mechanism for vascular enrichment.

We examined TSP1 expression in high grade and high stage human BC. Campbell *et al*. [31] showed reduced TSP1 expression in both high and low grade BC and suggested that the switch from an anti-angiogenic to angiogenic phenotype occurs early in BC development. The data suggest that down-regulation of TSP1 secretion in bladder is important in facilitating this switch. In addition, prostate biopsies indicated that patients with metastatic prostate cancer had significantly less TSP1 expression than patients with localized prostate cancer, who also had significantly lower TSP1 expression than patients with benign prostatic hyperplasia [48]. We examined the protein expression of TSP1 in human BC paired with normal bladder tissue from the same patients and found that the ~ 50 kDa TSP1 anti-angiogenic fragment is decreased in human urothelial cancer tissue. Eight of the ten paired samples had greater TSP1 protein expression in the normal tissue compared to the BC. One paired sample did not express TSP1 in either the normal or tumor tissue, and one paired sample showed higher TSP1 expression in the BC rather than the normal tissue. Previous immunohistochemical analysis of human BC indicated that poorly differentiated tumors expressed lower levels of TSP1 [49] and that tumors expressing less TSP1 at presentation were more likely to become invasive and/or metastatic [30]. Grossfeld *et al*. [29] found that patients with low TSP1 expression in BC had an increased risk of recurrence and decreased survival and that the expression of TSP1 was dependent on p53 expression: 96% of tumors with wildtype p53 expressed moderate to high levels of TSP1 protein, and only 58% of tumors with altered p53 protein overexpressed TSP1.

We do not mean to overstate the role of TSP1 in this mouse model of BC. The data presented here are mainly correlative, and, certainly, more experimentation to examine the mechanisms involved in androgen regulation of TSP1 is required. Furthermore, it is not our intention to assert that TSP1 is the only protein involved in this complex tumor model. Rather, TSP1 expression was investigated since it has previously been shown to be hormonally-regulated and plays a negative role in angiogenesis. Hormonal manipulation, specifically castration, can alter the expression of a host of other proteins involved in proliferation and/or angiogenesis. This report presents data that support an androgen-regulated TSP1 hypothesis, which may be important in the angiogenic capabilities of BC.

The UPII-SV40T model system employed here bypasses the need for a chemical carcinogen, which confounds analysis of androgen effects, and FPDCT allows longitudinal monitoring of tumor growth. Androgens increase tumor cell growth *in vitro *and *in vivo *and decrease TSP1 expression, possibly explaining the therapeutic effect of castration. This effect was seen even in cellular targets lacking detectable AR, indicating a stimulatory effect for androgens that may work through mechanisms other than AR e.g. other steroid receptors or non-steroid receptor mechanisms. Androgen stimulation may contribute to the increased incidence of BC in men, and androgenic suppression may be an avenue for BC prevention therapy via anti-androgens, LHRH agonists, and/or 5-alpha-reductase inhibitors. Others have reported changes in TSP1 expression in response to androgens in prostate and breast cancer. This report is the first to show modulation of TSP1 by androgen manipulation in a non-hormonal cancer.

## Conclusion

The UPII-SV40T model system employed here bypasses the need for a chemical carcinogen, which confounds analysis of androgen effects, and FPDCT allows longitudinal monitoring of tumor growth. Androgens increase tumor cell growth *in vitro *and *in vivo *and decrease TSP1 expression, possibly explaining the therapeutic effect of castration. This effect was seen even in cellular targets lacking detectable AR, indicating a stimulatory effect for androgens that may work through mechanisms other than AR e.g. other steroid receptors or non-steroid receptor mechanisms. Androgen stimulation may contribute to the increased incidence of BC in men, and androgenic suppression may be an avenue for BC prevention therapy via anti-androgens, LHRH agonists, and/or 5-alpha-reductase inhibitors. Others have reported changes in TSP1 expression in response to androgens in prostate and breast cancer. This report is the first to show modulation of TSP1 by androgen manipulation in a non-hormonal cancer.

## Competing interests

The authors declare that they have no competing interests.

## Authors' contributions

AMJ performed all animal manipulations, analyzed FPDCT data, carried out all *in vitro *work, performed protein expression assays and immunohistochemical staining, and drafted the manuscript. MJO assisted in all animal work and collected *in vitro *samples. JH performed immunohistochemical staining, collected and provided information on clinical samples. AMJ, HM, JLY, EMM, and JER conceived of different aspects of the study. AMJ, EMM, and JER participated in the design of the study. All authors read, critically edited for intellectual content, and approved the final manuscript.

## Pre-publication history

The pre-publication history for this paper can be accessed here:



## References

[B1] Madeb R, Messing EM (2004). Gender, racial and age differences in bladder cancer incidence and mortality. Urol Oncol.

[B2] American Cancer Society (2007). Cancer facts and figures 2007. American Cancer Society, Atlanta.

[B3] SEER Public-Use 1973–2002 – ASCII Text Data: (Surveillance, Epidemiology, and End Results (SEER) Program Public-Use Data (1973–2002), National Cancer Institute, DCCPS, Surveillance Research Program, Cancer Statistics Branch, released April 2005, based on the November 2004 submission. http://www.seer.cancer.gov.

[B4] McGrath M, Michaud DS, De Vivo I (2005). Hormonal and reproductive factors and risk of bladder cancer in women. Am J of Epidemiol.

[B5] Imaoka S, Yoneda Y, Sugimoto T, Hiroi T, Yamamoto K, Nakatani T, Funae Y (2000). CYP4B1 is a possible risk factor for bladder cancer in humans. Biochem Biophys Res Commun.

[B6] Imaoka S, Yoneda Y, Sugimoto T, Ikemoto S, Hiroi Y, Yamamoto K, Nakatani T, Funae Y (2001). Androgen regulation of CYP4B1 responsible for mutagenic activation of bladder carcinogens in the rat bladder: detection of CYP4B1 mRNA by competitive reverse transcription-polymerase chain reaction. Cancer Lett.

[B7] Isern J, Meseguer A (2003). Hormonal regulation and characterization of the mouse *Cyp4b1 *gene 5'-flanking region. Biochem Biophys Res Commun.

[B8] Ma J, Graves J, Bradbury JA, Zhao Y, Swope DL, King L, Wei Q, Clark J, Myers P, Walker V, Lindzey J, Korach KS, Zeldin DC (2004). Regulation of mouse renal CYP2J5 expression by sex hormones. Mol Pharmacol.

[B9] Tang W, Norlin M, Wikvall K (2007). Regulation of human CYP27A1 by estrogens and androgens in HepG2 and prostate cells. Arch Biochem Biophys.

[B10] Bertram JS, Craig AW (1972). Specific induction of bladder cancer in mice by butyl-(4-hydroxybutyl)-nitrosamine and the effects of hormonal modifications on the sex difference in response. Eur J Cancer.

[B11] Kono N, Tanahashi T, Suzawa N, Azuma C (1977). Effects of sex hormones on oncogenesis in rat urinary bladder by N-butyl-N-(4-hydroxybutyl)-nitrosamine. Int J Clin Pharmacol.

[B12] Imada S, Akaza H, Ami Y, Koiso K, Ideyama Y, Takenaka T (1997). Promoting effects and mechanisms of action of androgen in bladder carcinogenesis in male rats. Eur Urol.

[B13] Miyamoto H, Yang Z, Chen YT, Ishiguro H, Uemura H, Kubota Y, Nagashima Y, Chang YJ, Hu YC, Tsai MY, Yeh S, Messing EM, Chang C (2007). Promotion of bladder cancer development and progression by androgen receptor signals. J Natl Cancer Inst.

[B14] Zhang ZT, Pak J, Shapiro E, Sun TT, Wu XR (1999). Urothelium-specific expression of an oncogene in transgenic mice induced the formation of carcinoma in situ and invasive transitional cell carcinoma. Cancer Res.

[B15] Baenziger NL, Brodie GN, Majerus PW (1971). A thrombin-sensitive protein of human platelet membranes. Proc Natl Acad Sci USA.

[B16] Lawler J, Slayter HS, Coligan JE (1978). Isolation and characterization of a high molecular weight glycoprotein from human platelets. J Biol Chem.

[B17] Majack RA, Goodman LV, Dixit VM (1988). Cell surface thrombospondin is functionally essential for vascular smooth muscle cell proliferation. J Cell Biol.

[B18] Jaffe EA, Ruggiero JT, Falcone DJ (1985). Monocytes and macrophages synthesize and secrete thrombospondin. Blood.

[B19] Mosher DF, Doyle MJ, Jaffe EA (1982). Synthesis and secretion of thrombospondin by cultured human endothelial cells. J Cell Biol.

[B20] Raugi GJ, Mumby SM, Abbott-Brown D, Bornstein P (1982). Thrombospondin: synthesis and secretion by cells in culture. J Cell Biol.

[B21] Adams JC (2001). Thrombospondins: multifunctional regulators of cell interactions. Annu Rev Cell Dev Biol.

[B22] Adams JC, Monk R, Taylor AL, Ozbek S, Fascetti N, Baumgartner S, Engel J (2003). Characterisation of *drosophila *thrombospondin defines an early origin of pentameric thrombospondins. J Mol Biol.

[B23] Armstrong LC, Bornstein P (2003). Thrombospondins 1 and 2 function as inhibitors of angiogenesis. Matrix Biol.

[B24] Lawler J, Detmar M (2004). Tumor progression: the effects of thrombospondin-1 and -2. Int J Biochem Cell Biol.

[B25] Adams JC, Lawler J (2004). The thrombospondins. Int J Biochem Cell Biol.

[B26] Tolsma SS, Volpert OV, Good DJ, Frazier WA, Polverini PJ, Bouck N (1993). Peptides derived from two separate domains of the matrix protein thrombospondin-1 have anti-angiogenic activity. J Cell Biol.

[B27] Dawson DW, Pearce SFA, Zhong R, Silverstein RL, Frazier WA, Bouck NP (1997). CD36 mediates the in vitro inhibitory effects of thrombospondin-1 on endothelial cells. J Cell Biol.

[B28] Jimenez B, Volpert OV, Crawford SE, Febbraio M, Silverstein RL, Bouck N (2000). Signals leading to apoptosis-dependent inhibition of neovascularization by thrombospondin-1. Nat Med.

[B29] Grossfeld GD, Ginsberg DA, Stein JP, Bochner BH, Esrig D, Groshen S, Dunn M, Nichols PW, Taylor CR, Skinner DG, Cote RJ (1997). Thrombospondin-1 expression in bladder cancer: association with p53 alterations, tumor angiogenesis, and tumor progression. J Natl Cancer Inst.

[B30] Goddard JC, Sutton CD, Jones JL, O'Byrne KJ, Kockelbergh RC (2002). Reduced thrombospondin-1 at presentation predicts disease progression in superficial bladder cancer. Eur Urol.

[B31] Campbell SC, Volpert OV, Ivanovich M, Bouck NP (1998). Molecular mediators of angiogenesis in bladder cancer. Cancer Res.

[B32] Colombel M, Filleur S, Fournier P, Merle C, Guglielmi J, Courtin A, Degeorges A, Serre CM, Bouvier R, Clézardin P, Cabon F (2005). Androgens repress the expression of the angiogenesis inhibitor thrombospondin-1 in normal and neoplastic prostate. Cancer Res.

[B33] Mattila MM, Tarkkonen KM, Seppäne JA, Ruohola JK, Valve EM, Härkönen PL (2006). Androgen and fibroblast growth factor 8 (FGF8) downregulation of thrombospondin 1 (TSP1) in mouse breast cancer cells. Mol Cell Endocrinol.

[B34] Ning R, Tang X, Conover D, Yu R (2003). Flat panel detector-based cone beam computed tomography with a circle-plus-two-arcs data acquisition orbit: preliminary phantom study. Med Phys.

[B35] Conover DL, Ning R, Yu R, Lu X, Wood RW, Reeder JE, Johnson AM (2005). Small animal imaging using a flat panel detector-based cone beam computed tomography (FPD-CBCT) imaging system. Proc SPIE Med Imaging.

[B36] Johnson AM, Conover DL, Huang J, Messing EM, Ning R, O'Connell MJ, Rossi MA, Sun TT, Wood RW, Wu XR, Reeder JE (2006). Early detection and measurement of urothelial tumors in mice. Urology.

[B37] Southgate J, Harnden P, Trejdosiewicz LK (1999). Cytokeratin expression patterns in normal and malignant urothelium: a review of the biological and diagnostic implications. Histol Histopathol.

[B38] Drews U, Sulak O, Oppitz M (2001). Immunohistochemical localization of androgen receptor during sex-specific morphogenesis in the fetal mouse. Histochem Cell Biol.

[B39] Takeda H, Mizuno T, Lasnitzki I (1992). Autoradiographic studies of androgen-binding sites in the rat urogenital sinus and postnatal prostate. Gynecol Obstet Invest.

[B40] Bentvelsen FM, McPhaul MJ, Wilson JD, George FW (1994). The androgen receptor of the urogenital tract of the fetal rat is regulated by androgen. Mol Cell Endocrinol.

[B41] Salmi S, Santti R, Gustafsson J, Makela S (2001). Co-localization of androgen receptor with estrogen receptor β in the lower urinary tract of the male rat. J Urol.

[B42] Shapiro E, Huang HY, Wu XR (2000). Uroplakin and androgen receptor expression in the human fetal genital tract: insights into the development of the vagina. J Urol.

[B43] Zhuang YH, Blauer M, Temmela T, Tuohimaa P (1997). Immunodetection of androgen receptor in human urinary bladder cancer. Histopathology.

[B44] Laor E, Schiffman ZJ, Braunstein JD, Reid RE, Tolia BM, Koss LG, Freed SZ (1985). Androgen receptors in bladder tumors. Urology.

[B45] Noronha RFX, Rao BR (1986). Sex hormone receptors in localized and advanced transitional cell carcinoma of urinary tract in humans. Urology.

[B46] Boorjian S, Ugra S, Mongan NP, Gudas NJ, You X, Tickoo SK, Scherr DS (2004). Androgen receptor expression is inversely correlated with pathologic tumor stage in bladder cancer. Urology.

[B47] Guerini V, Sau D, Scaccianoce E, Rusmini P, Ciana P, Maggi A, Martini PG, Katzenellenbogen BS, Martini L, Motta M, Poletti A (2005). The androgen derivative 5α-androstane-3β,17β-diol inhibits prostate cancer cell migration through activation of the estrogen receptor β subtype. Cancer Res.

[B48] Kwak C, Jin RJ, Lee C, Park MS, Lee SE (2002). Thrombospondin-1, vascular endothelial growth factor expression and their relationship with p53 status in prostate cancer and benign prostatic hyperplasia. BJU Intl.

[B49] Ioachim E, Michael MC, Salmas M, Damala K, Tsanou E, Michael MM, Malamou-Mitsi V, Stavropoulos NE (2006). Thrombospondin-1 expression in urothelial carcinoma: prognostic significance and association with p53 alterations, tumour angiogenesis and extracellular matrix components. BMC Cancer.

